# Supplementary material for: Increased Gene Targeting in Hyper-Recombinogenic LymphoBlastoid Cell Lines Leaves Unchanged DSB Processing by Homologous Recombination
Source: Int J Mol Sci. 2022 Aug 16;23(16):9180. doi: 10.3390/ijms23169180 (PMC9409177; doi:10.3390/ijms23169180)
Supplement: Supplementary file 1 [file ijms-23-09180-s001.zip › ijms-1829098-supplementary.pdf]

Increased gene-targeting in hyper-recombinogenic lymphoblastoid cell lines leaves unchanged DSB processing by homologous recombination

Emil Mladenov, Katja Paul-Konietzko, Veronika Mladenova, Martin [3] Stuschke, and George Iliakis

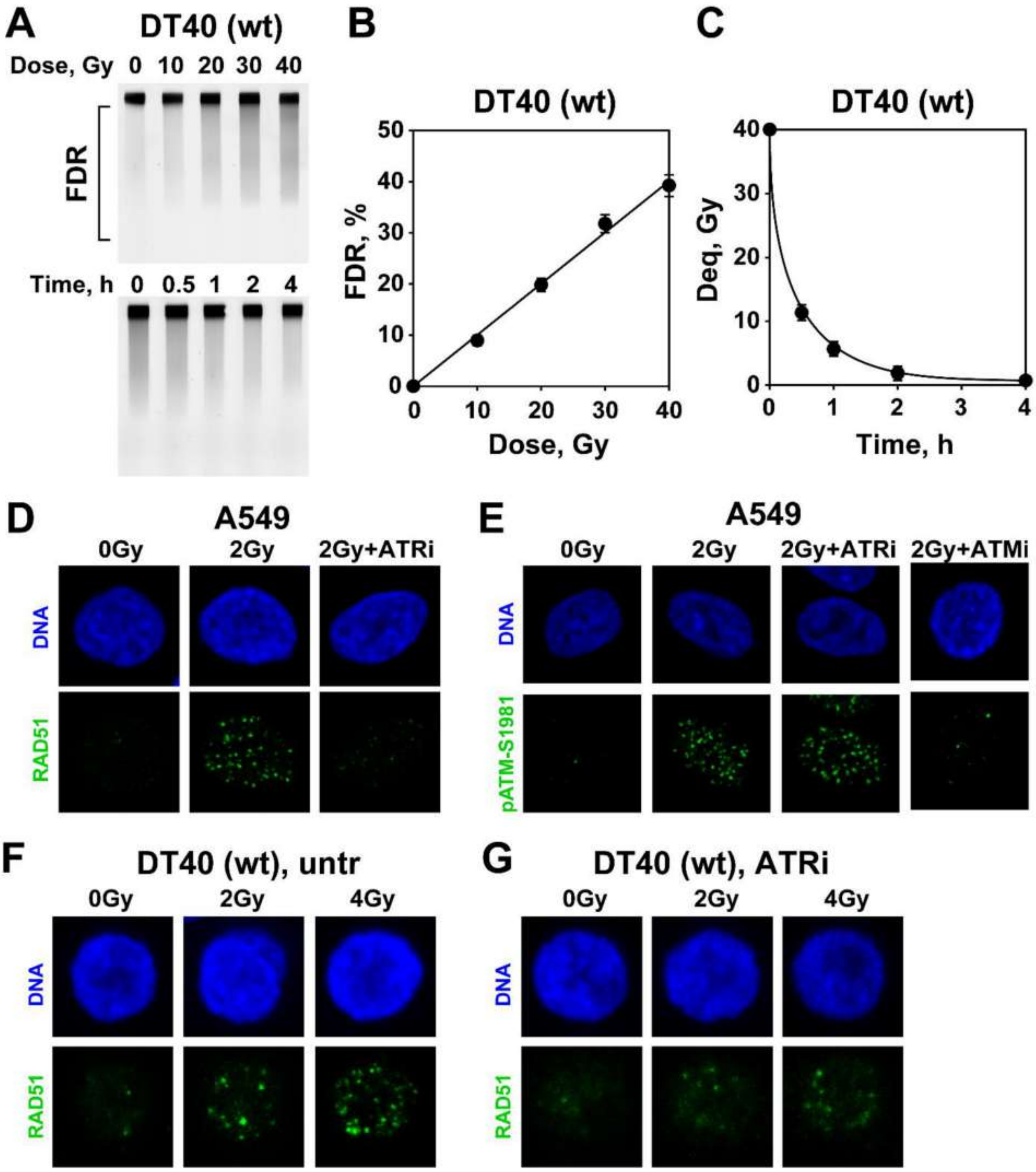

Figure S1. VE-821 is a potent inhibitor of HR: (**A**) Representative images of PFGE gels showing the dose response of DT40 cells exposed to increasing IR doses (0-40 Gy, upper panel) and repair kinetics of DT40 cells exposed to 40 Gy, lower panel; (**B**) Quantitative densitometry analysis of PFGE gels shown in Figure S1A, upper panel; (**C**) Quantitative densitometry analysis of PFGE gels shown in Figure S1A, lower panel; (**D**) Representative IF images of RAD51 foci in A549 cells exposed to 2 Gy in the presence of ATRi. (**E**) Representative IF images of ATM-pS1981 foci in A549 cells exposed to 2 Gy in the presence of ATRi or ATMi. (**F**) Representative IF images of RAD51 foci in untreated DT40 cells. (**G**) Same as Figure S1F, but for DT40 cells treated with ATRi.

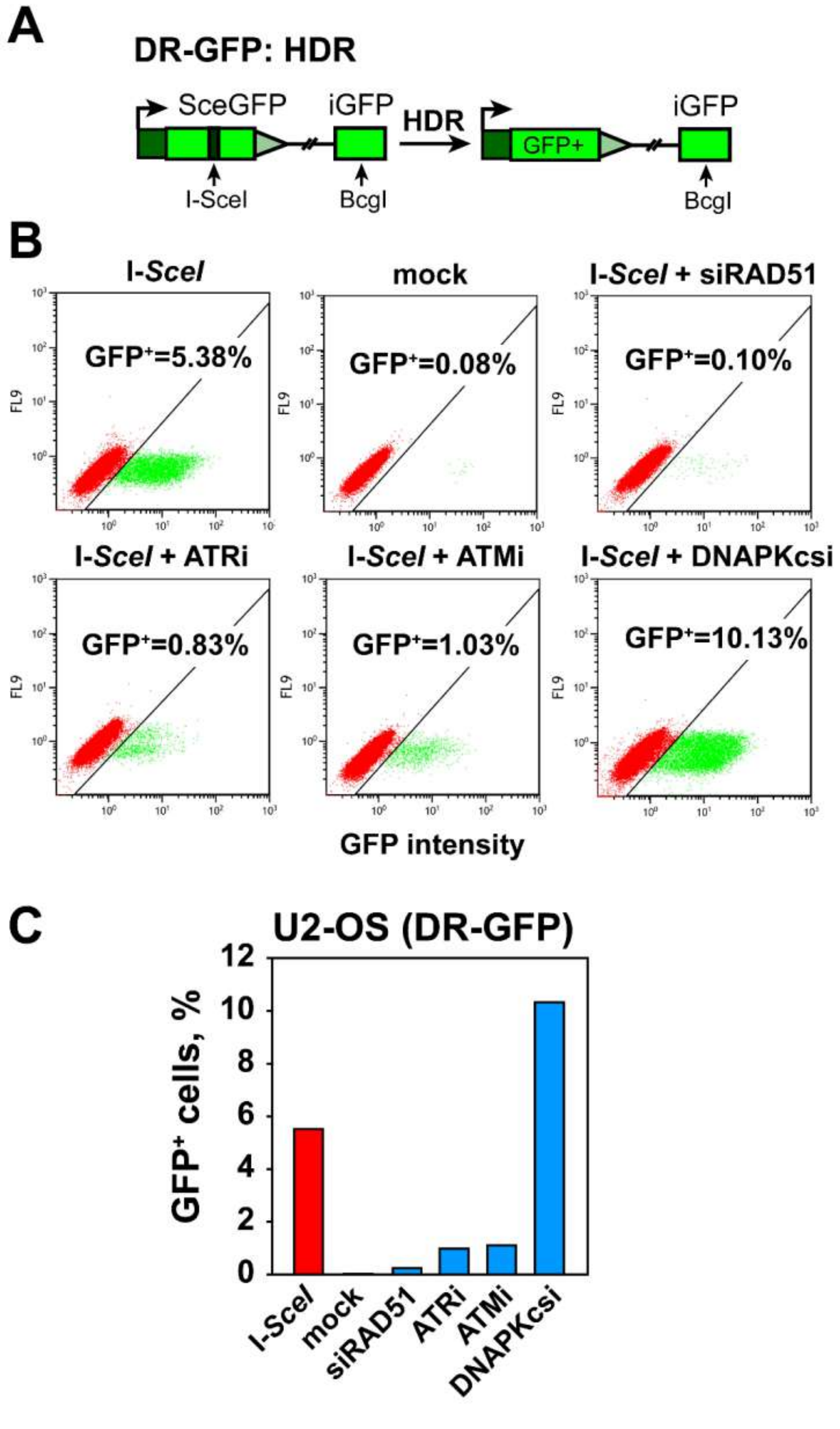

Figure S2. VE-821 inhibits HR: (**A**) Schematic representation of the DR-GFP construct integrated in human U-2 OS cells, for the quantification of HR; (**B**) Dot Plots of GFP expression in U-2 OS cells after transfection with an I-*SceI* expressing plasmid at the indicated conditions. (**C**) Bar plots showing the number of GFP-positive cells, measured by flow cytometry at the conditions indicated in Figure S2B.

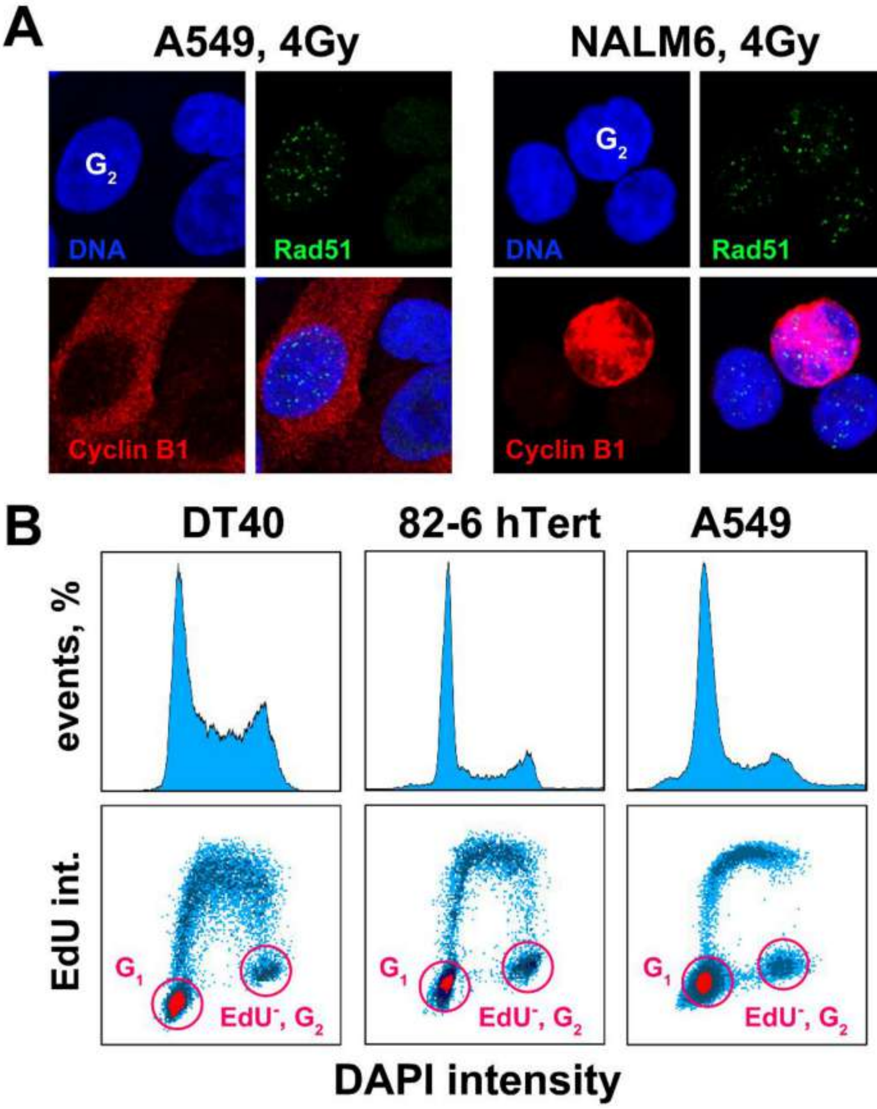

Figure S3. Cell cycle specific determination of IR-induced foci: (**A**) Representative IF images showing the specificity and intensity of Cyclin B1 staining in A549 and NALM6 cells. Cells identified as G_2-cells are indicated; (**B**) Histogram and dot plots showing the distribution of cells throughout the cell cycle, as well as the EdU intensity in DT40, 82-6 hTert and A549 cells. DAPI and EdU intensity were the parameters used in the QIBC analysis, to trace for G_1- or G_2-cells. The gates defining cells in G_1-phase and G_2-phase are depicted as red circles.

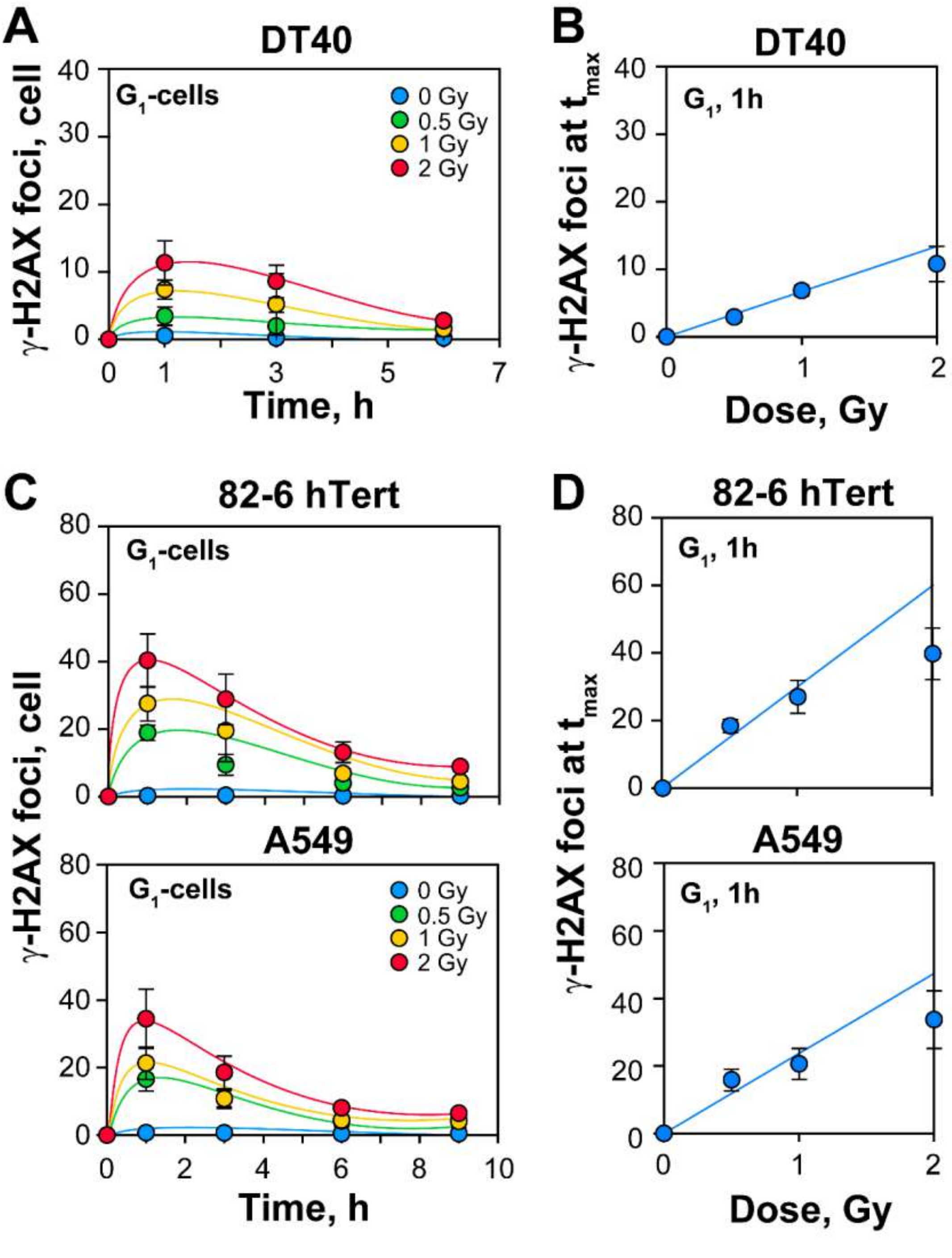

Figure S4. Repair kinetics and dose response curves of γ-H2AX foci in G1-phase cells: (**A**) γ-H2AX repair kinetics of DT40 cells in G_1-phase of the cell cycle. Cells are selected on the basis of the gates depicted in Figure S3B; (**B**) Dose response of γ-H2AX foci in G_1-phase DT40 cells; (**C**) Same as Figure S4A, but for 82-6 hTert and A549 cells; (**D**) Same as Figure S4B, but for 82-6 hTert and A549 cells. Data represent mean and standard deviation from three independent experiments.

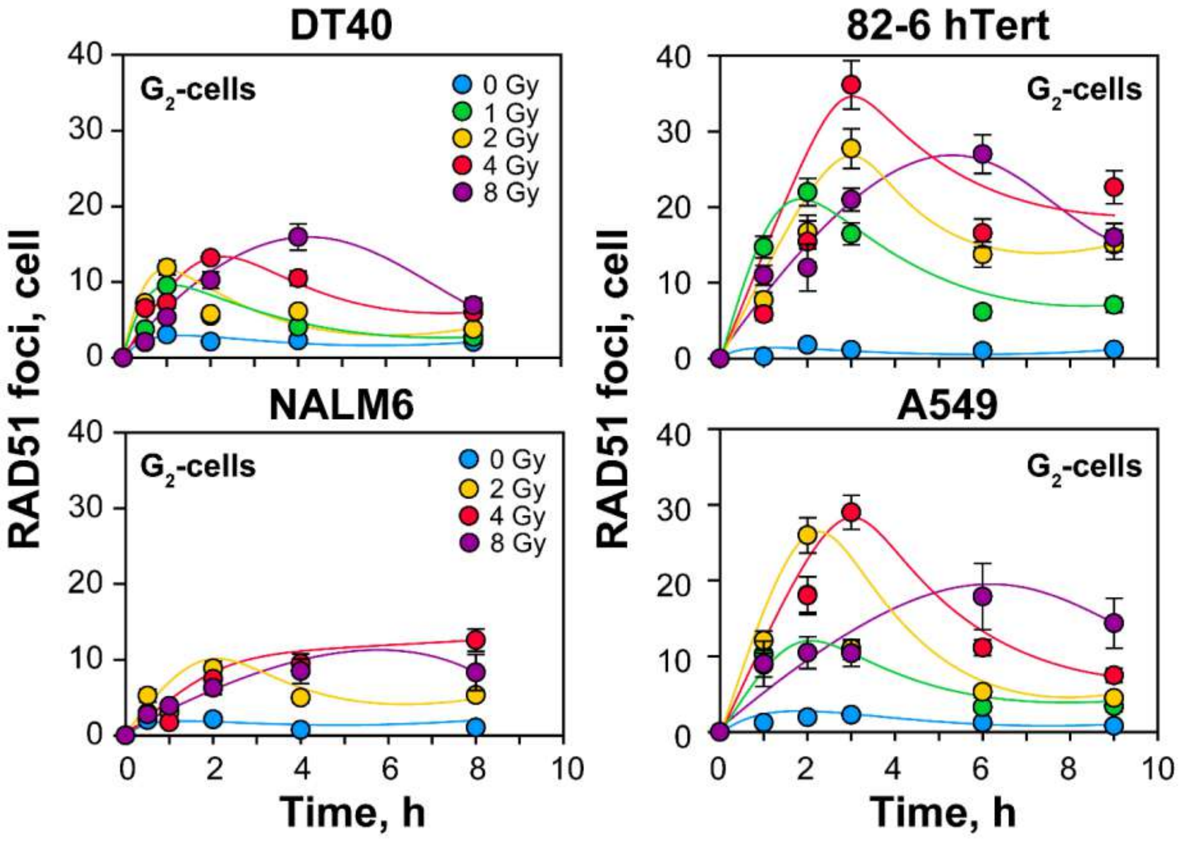

Figure S5. RAD51 foci formation and decay in DT40, NALM6, 82-6 hTert and A549 cells, irradiated in G_2-phase of the cell cycle: The generated data was used to plot the dose response curves of RAD51 foci formation at t_{max} in Figure 3. Data represent mean and standard deviation from three independent experiments.

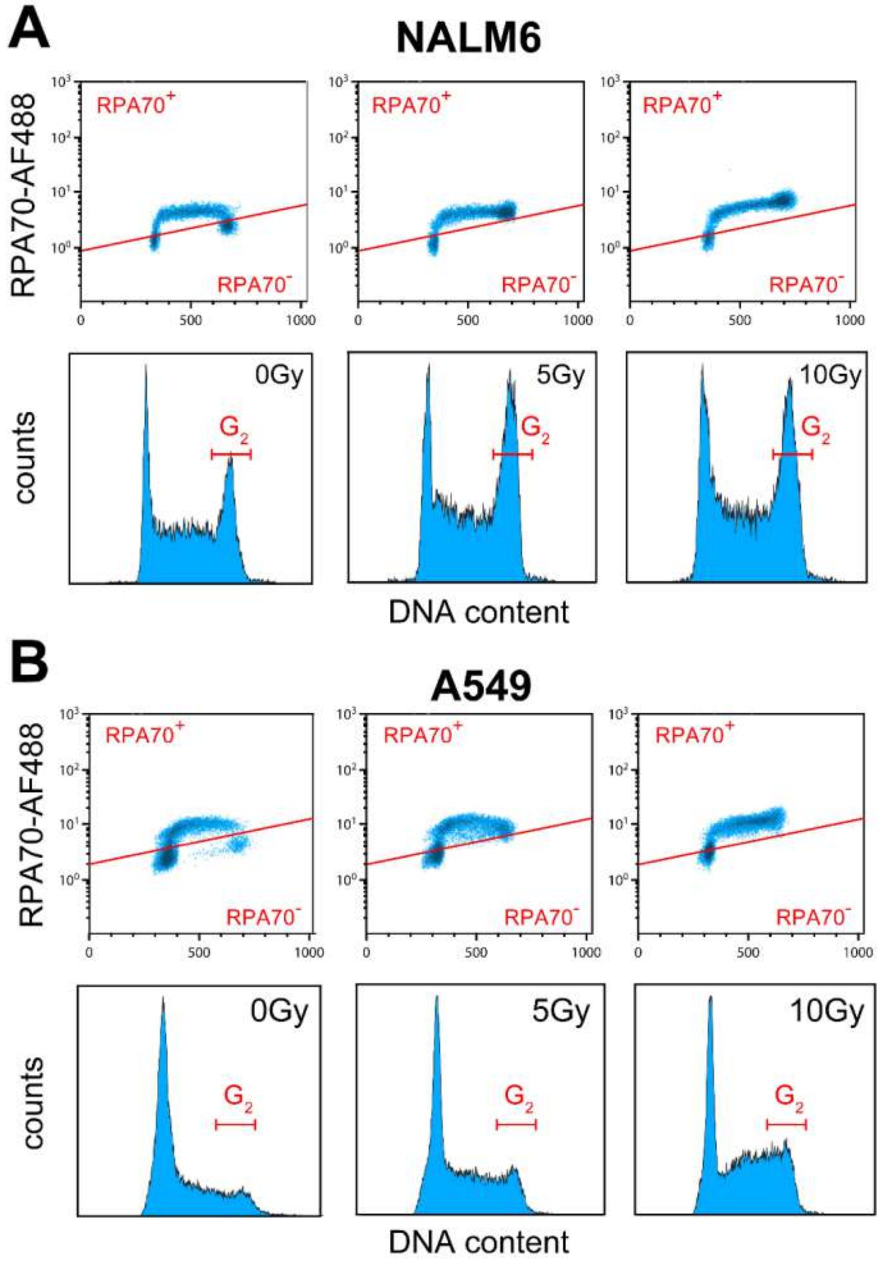

Figure S6. Analysis of DNA end-resection in G_2-phase cells: (**A**) Dot and histogram plots, illustrating the RPA70 fluorescence intensity as a function of IR dose in NALM6 cells. The DNA content is measured by propidium iodide (PI) staining and the histogram plots show the distribution of cells through the cells cycle at the time of RPA70 signal determination. The G_2-gates used to determine the RPA70 signal are indicated as red line and represents the fraction of cells in G_2-phase, determined by the PI staining; (**B**) Same as Figure S6A, but for A549 cells.

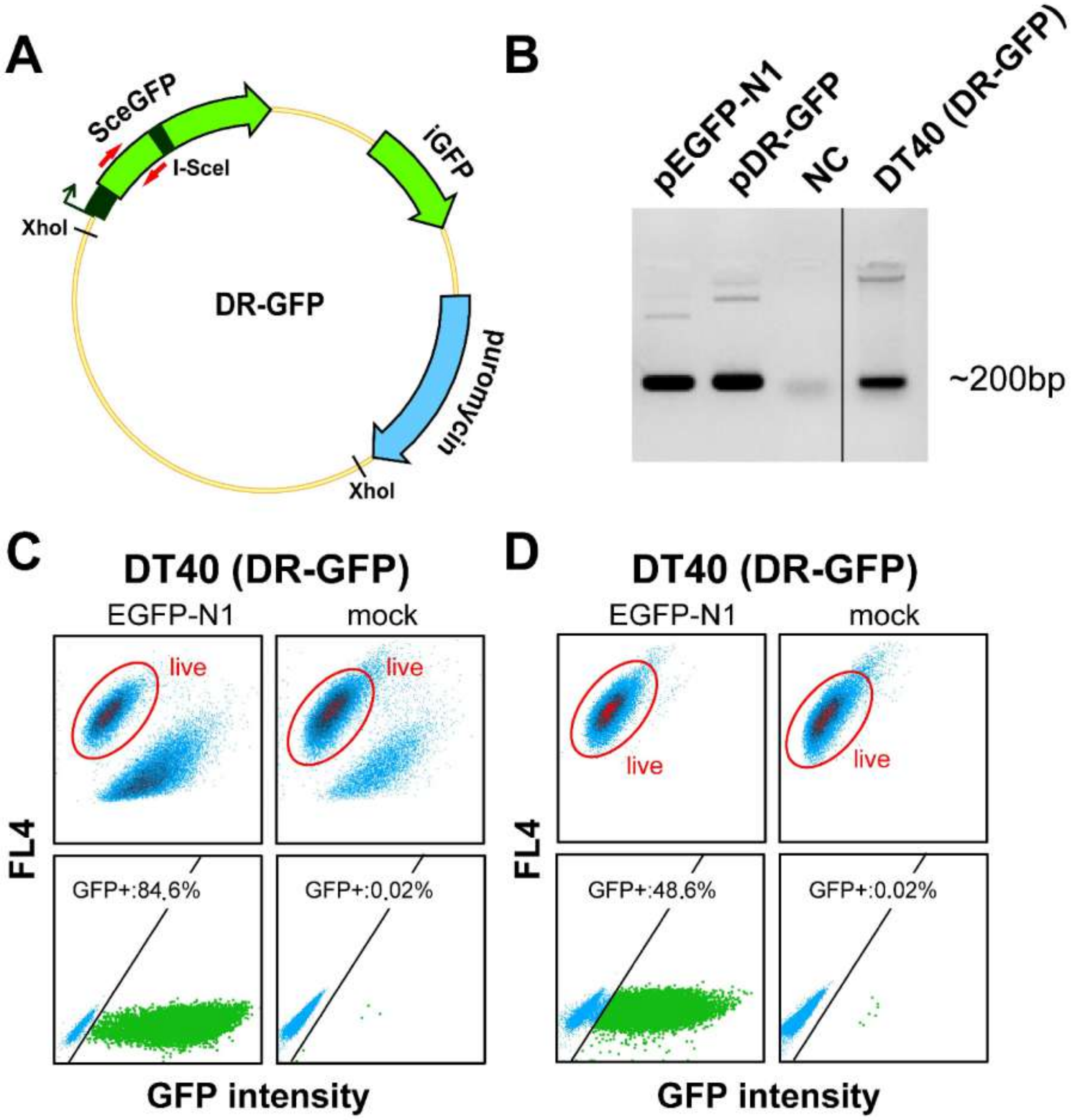

Figure S7. Generation of DR-GFP DT40 cells: (**A**) Schematic representation of the DR-GFP vector integrated into the genome of DT40 cells for quantification of HR efficiency. The essential elements of the vector (DR-GFP construct, puromycin selection, etc.) are shown. Before transfection, the plasmid was linearized at the XhoI site and only the essential part of the construct was utilized for integration. The positive DT40 clones carrying the DR-GFP cassette were identified by PCR using the indicated primers. The PCR positive clones were further selected by puromycin treatment. (**B**) Agarose gels, showing the product of the PCR reactions with the following DNA templates: pEGFP-N1, pDR-GFP, negative control (NC) and genomic DNA from the selected DT40 clone. (**C**) Representative dot plots of the DT40 DR-GFP clone transfected with pEGFP-N1 for the determination of transfection efficiency. A transfection program optimized for maximum transfection efficiency was used. Non-transfected cells (mock) were used as a control to show the level of spontaneous generation of GFP positive cells in the DT40 DR-GFP clone. (**D**) Same as in Figure S7C, but for transfection with a program optimized for maximum survival.

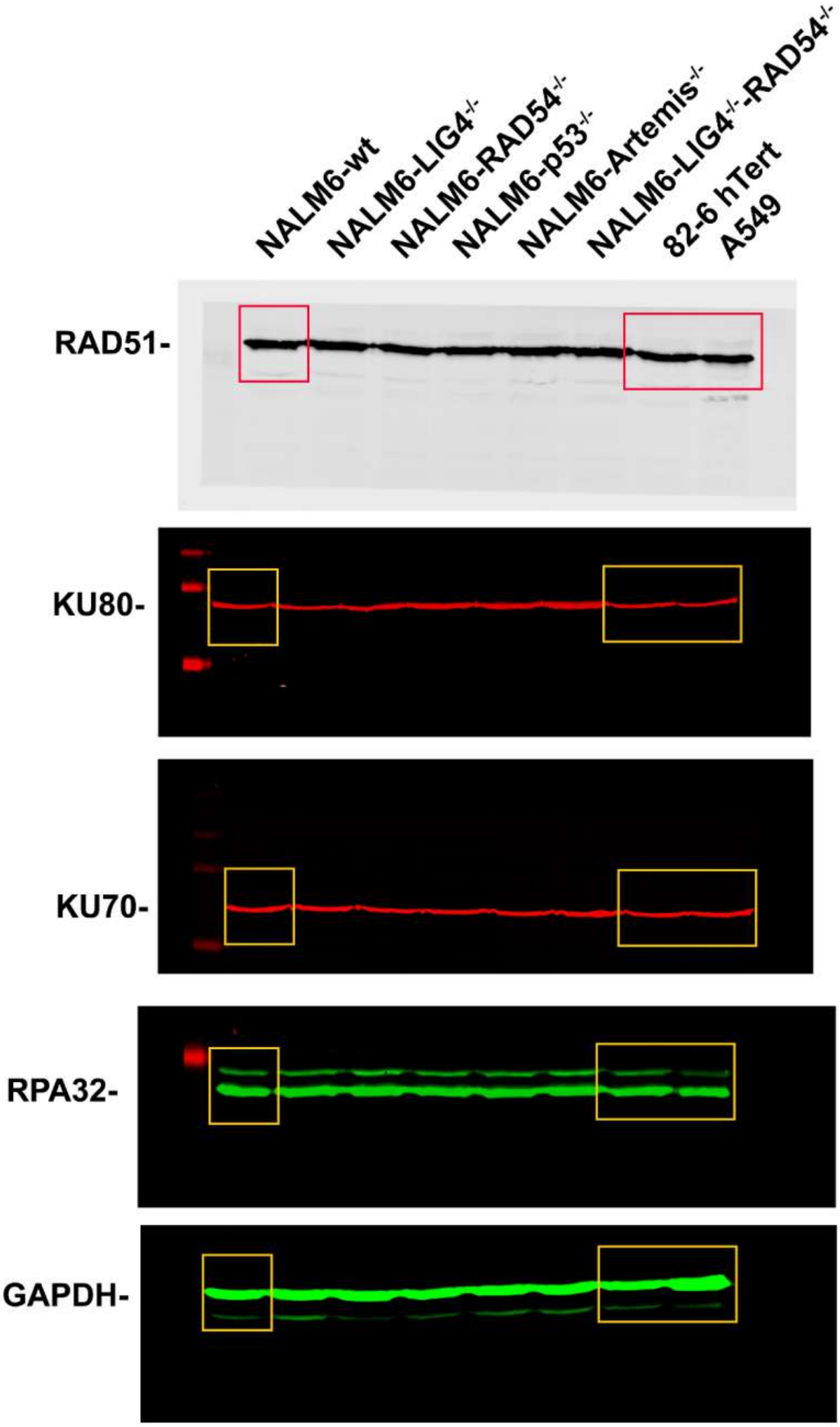

Figure S8 RAW files of Western blot membrane scans used to generate the images shown in Figure 5C. The cropped lanes are illustrated as red or yellow colored rectangles.
